# Syphilitic Uveitis With Diverse Clinical Presentations: Multimodal Imaging as a Useful Adjunctive Tool for Diagnosis and Treatment

**DOI:** 10.7759/cureus.59791

**Published:** 2024-05-07

**Authors:** Saori Kawamoto, Tomona Hiyama, Ikuyo Sada, Yosuke Harada

**Affiliations:** 1 Ophthalmology, Hiroshima Prefectural Hospital, Hiroshima, JPN; 2 Ophthalmology, Hiroshima University, Hiroshima, JPN

**Keywords:** multimodal imaging, acute syphilitic posterior placoid chorioretinitis, chorioretinitis, uveitis, syphilis

## Abstract

We report four cases of syphilitic uveitis with diverse clinical presentations. All patients were men who have sex with women, and were aged 19-68 years, and none were HIV-positive. All cases were bilateral. One case presented with anterior uveitis, while three exhibited panuveitis. One patient had acute syphilitic posterior placoid chorioretinitis and two had retinal vasculitis resulting in damage to the outer retinal and retinal pigment epithelium. The rapid plasma reagin (RPR) test and *Treponema pallidum *(TP) hemagglutination test were both positive in all cases. Six of eight eyes had improved vision and best-corrected visual acuity better than 20/20 after antibiotic treatment. Serological testing is mandatory for the diagnosis of syphilitic uveitis. Additionally, multimodal imaging, including optical coherence tomography (OCT), fundus autofluorescence (FAF), and fluorescein angiography (FA), can provide useful adjunctive information for early diagnosis and assessment of treatment response.

## Introduction

Syphilis is a sexually transmitted infection that has periodic outbreaks worldwide [1]. In Japan, the number of infected individuals has been increasing rapidly since 2012 [2], with 13,228 reported cases of syphilis as of 2022 [3]. Ocular syphilis, often referred to as the "great imitator", is associated with diverse clinical courses [4]. It can manifest as bilateral or unilateral, granulomatous or non-granulomatous, and affect the anterior, posterior, or both regions. These varied presentations make its diagnosis based on clinical progression challenging [5]. We present four cases of syphilitic uveitis with varying clinical presentations, evaluated using multiple imaging modalities such as optical coherence tomography (OCT), fluorescein angiography (FA), or fundus autofluorescence (FAF).

## Case presentation

Case 1

A 19-year-old male presented with conjunctival congestion and pain in his right eye. The patient was diagnosed with bilateral iritis with peripheral anterior synechia in the right eye. His symptoms did not improve despite treatment with antibiotics and steroid eye drops for one week, and the condition of the left eye worsened; therefore, the patient was referred to the Ophthalmology Department at Hiroshima University Hospital.

**Figure 1:**
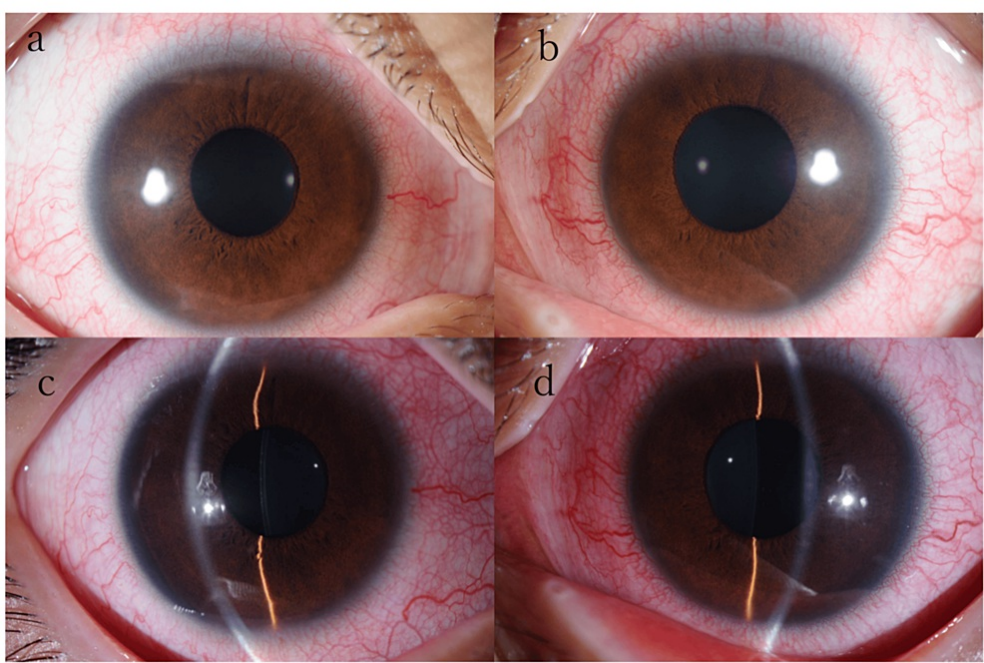
Clinical appearance of the right eye (a, c) and left eye (b, d) of Case 1 at presentation Slit-lamp examination showed ciliary injection and pigmented keratic precipitates in both eyes, predominantly in the left eye

While the patient had no significant medical history, he had engaged in sexual activity with a sex worker one year prior. Best-corrected visual acuity was 20/20 in both eyes, and intraocular pressure in the right and left eyes was 18 and 10 mmHg, respectively. Slit-lamp examination showed ciliary injection and pigmented keratic precipitates in both eyes (predominantly in the left eye) (Figure 1).

Anterior chamber cell grade 2+ was observed in the right eye, while grade 3+ was noted in the left eye, as per the Standardization of Uveitis Nomenclature Working Group classification [6]. Laser flare photometry value [objective and quantitative measurement of aqueous humor protein levels in the anterior chamber where the physiological value is approximately 5 photon count per millisecond (ph/ms)] was 40.3 ph/ms in the right eye and 262.5 ph/ms in the left eye. No abnormal findings were noted by fundus examination. However, OCT showed hyper-reflective deposits with a vitreoretinal interface in the left eye. FA revealed leakage around the optic disc in the left eye and the peripheral area in both eyes. The rapid plasma reagin (RPR) test and anti-*Treponema pallidum* (TP) antibody were positive, leading to the diagnosis of syphilitic uveitis. HIV testing was negative. Serology for the hepatitis B virus (HBV) and the human T-lymphotropic virus type 1 (HTLV-1) testing were also negative. An ulcer suspected to be a chancre was found on the penis.

As the patient preferred outpatient treatment, he was started on oral amoxicillin 2000 mg/day and probenecid; 0.1% betamethasone sodium phosphate eye drops four times a day in both eyes were also initiated. Anterior chamber cells were reduced to grade 0.5+ in both eyes within a week. After four weeks of oral amoxicillin, the RPR test showed a decrease from 185-fold to 11.2-fold, and the treatment was terminated. One month after treatment, FA showed improvement in optic disc leakage and vasculitis, and OCT revealed the resolution of hyper-reflective deposits. The final best-corrective visual acuity was 20/15 in both eyes.

Case 2

**Figure 2:**
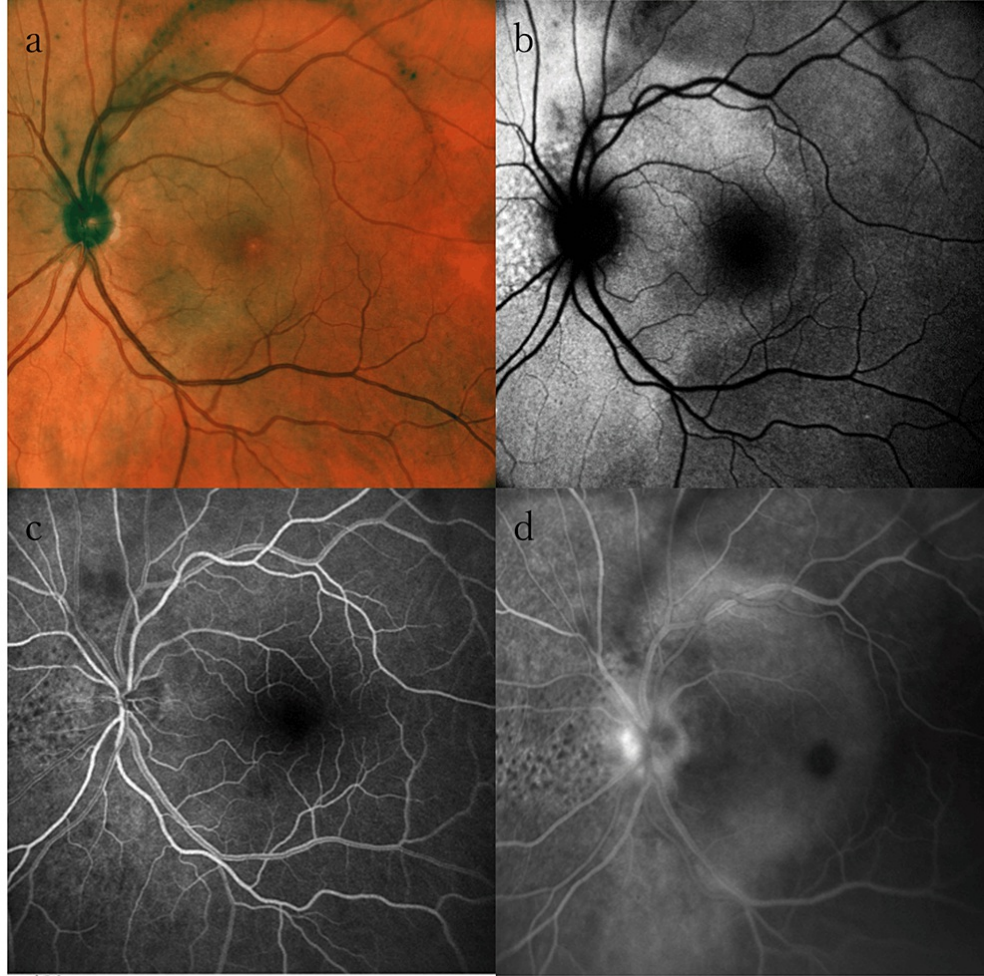
Optical findings in the left eye of Case 2 during the initial visit (a) Color fundus photography. Fundus examination revealed a yellow-white placoid retinal lesion encircling the optic disc and involving the macula in the left eye. Additionally, there was swelling in the optic disc. (b) FAF. The placoid lesion exhibited hyperfluorescence. Faint punctate lesions were observed on the nasal side of the optic disc. (c) and (d) FA in the early and late phases showed hyperfluorescence due to a window defect, along with leakage of fluorescence from the left optic disc. The nasal hypofluorescent punctate lesions observed on FA corresponded with the hyperfluorescent findings on FAF FA: fluorescein angiography; FAF: fundus autofluorescence

A 68-year-old male was admitted to our hospital with a complaint of decreased vision in the left eye for one week. He had a two-month history of oral ulcers and a one-month history of itchy erythematous patches spread throughout the body. He had engaged in sexual contact with his partner three months prior but had no history of involvement in sexual activity with sex workers or same-sex encounters. Visual acuity was 20/15 in the right eye and 20/250 in the left eye. Intraocular pressure was 16 mmHg in both eyes. The pupillary light reflex was slightly dull in the left eye. Goldmann visual field examination showed a central scotoma in the left eye. Slit-lamp examination showed grade 1+ cells in the anterior chamber and vitreous in both eyes. Fundus examination was unremarkable in the right eye, and a yellow-white placoid lesion was observed in the macular area of the left eye. FAF showed hyperfluorescence involving the macula in the left eye, corresponding to the placoid lesion revealed by fundus examination. FA of the left eye revealed hyperfluorescence caused by a window defect in the placoid lesion, along with fluorescence leakage from the optic disc. In the placoid area, punctate lesions were observed with hyperfluorescence on FAF and hypofluorescence on FA (Figure 2).

**Figure 3:**
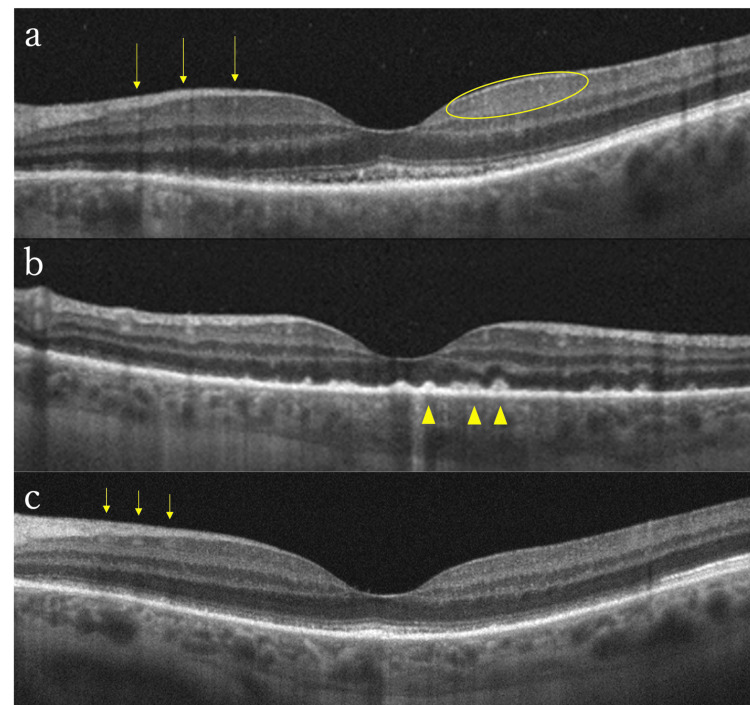
OCT findings in the left eye before and after antibiotic treatment in Case 2 (a) OCT at the first visit revealed linear hyper-reflective lesions from the nerve fiber layer to the inner plexiform layer (arrows) and tiny punctate hyper-reflective dots in the inner retinal layers (circle). There was subretinal fluid under the macula and inflammatory cells in the vitreous. (b) OCT after completion of 14 days of intravenous benzylpenicillin. EZ became indefinite and multiple nodular irregularities at the level of the RPE emerged (arrowheads). Tiny punctate hyper-reflective dots spread to the inner layer of the retina. (c) OCT two months after completion of antibiotic treatment. High reflective patchy lesions remained in the ganglion cell layer (arrows), but the segmental loss of the EZ and RPE irregularity were improved EZ: ellipsoid zone; OCT: optical coherence tomography; RPE: retinal pigment epithelium

OCT showed hyper-reflective lesions in the inner plexiform layer and the ganglion cell layer. The ellipsoid zone (EZ) was indistinct, and subretinal fluid was present beneath the macula (Figure 3).

Critical flicker fusion frequency, which is typically indicative of optic nerve function, measured at 43.5 Hz in the right eye and 12.5 Hz in the left eye (>35 Hz is considered normal, and <25 Hz is abnormal) [7], indicating optic neuropathy. Ulcers were present on the lips and pharynx. Laboratory tests revealed a positive RPR qualitative test and TP antibody, leading to a diagnosis of syphilitic uveitis. Serology for HIV, HBV, and HTLV-1 testing were negative. Intravenous administration of benzylpenicillin (PCG) (24 MU/day) for 14 days was started. At the end of the treatment, visual acuity improved to 20/100 in the left eye. OCT showed hyper-reflective nodular elevation on the retinal pigment epithelium (RPE) (Figure 3). At 47 days after treatment completion, visual acuity was 20/15 in the right eye and 20/20 in the left eye. Critical flicker fusion frequency in the left eye improved to 44.0 Hz, and the central scotoma in the left eye disappeared. Although the yellow-white lesions in the fundus and hyperfluorescence on FAF persisted, OCT did not show discontinuity of the EZ (Figure 3). The granular lesions inside the placoid area became more distinct during antibiotic treatment but became indistinct and faint two months after treatment completion. The RPR quantitative test showed a decrease from 80-fold before treatment to 3.2-fold.

Case 3

**Figure 4:**
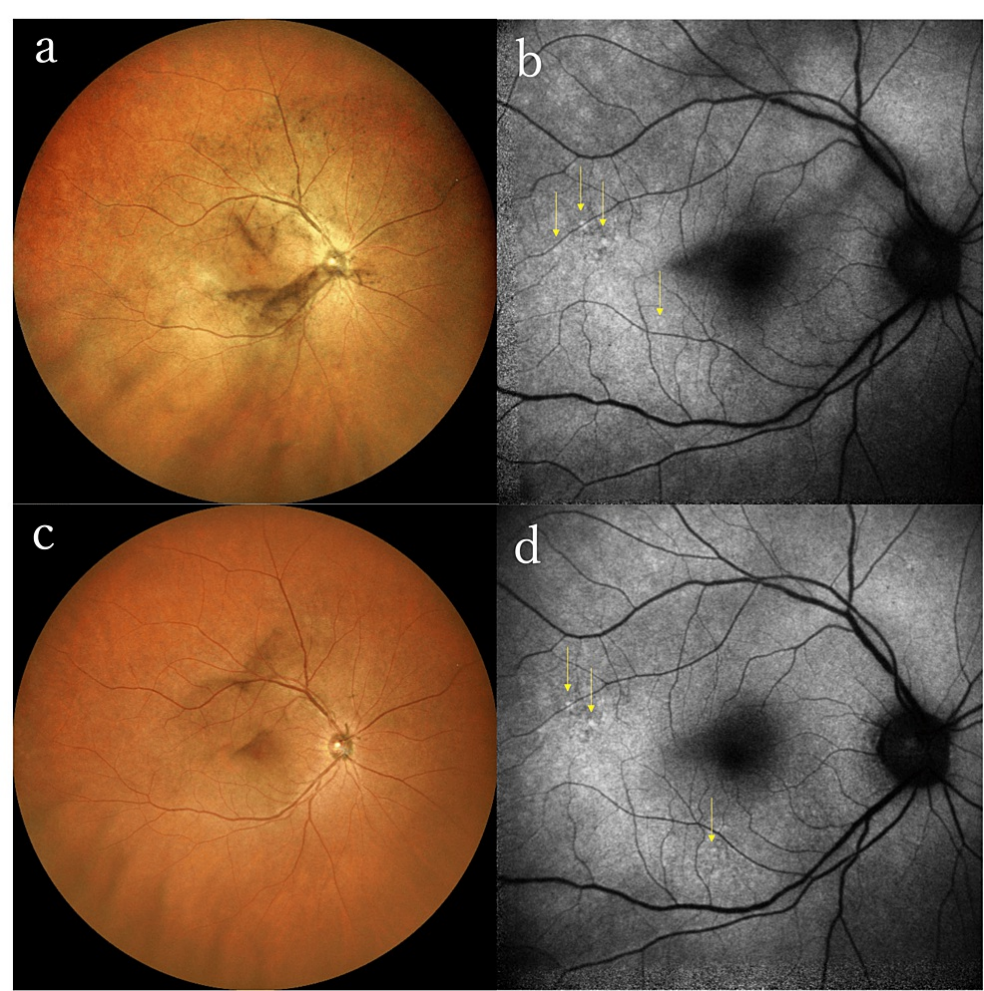
Color fundus photography and FAF of the right eye at presentation and post-treatment in Case 3 (a) Fundus examination at the initial visit showed vitreous opacity around the swollen disc and salt and pepper-like pigment deposits in the peripheral area. Retinal veins were irregularly dilated. (b) FAF revealed granular hyperfluorescence around the macula and arcade vessel (arrows), indicating retinal epithelial defect. (c) Color fundus photography after antimicrobial treatment showed improved vitreous haze and no pigment deposits. (d) FAF after treatment demonstrated persistent hyperfluorescence (arrows) FAF: fundus autofluorescence

A 60-year-old male with a history of ulcerative colitis, hypertension, and dyslipidemia sought medical attention for bilateral blurred vision and floaters that had persisted for two months. He was diagnosed with bilateral noninfectious uveitis and received a bilateral subtenon injection of triamcinolone acetonide. One month later, he developed a central scotoma in the right eye, leading to a referral to the Ophthalmology Department at Hiroshima University Hospital. At the initial visit, visual acuity was 20/200 in the right eye and 20/40 in the left eye. The intraocular pressure was 15 mmHg in both eyes. There were grade 0.5+ anterior chamber cells and grade 1+ vitreous haze in both eyes. Fundus examination revealed granular pigment deposits in both eyes, but there was no optic disc swelling, hyperemia, or yellow-white lesions (Figure 4).

**Figure 5:**
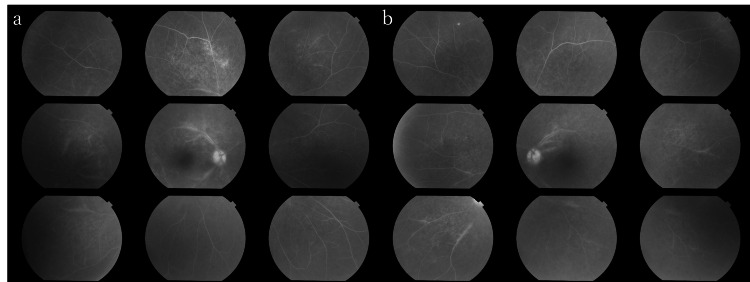
FA before the antibiotic treatment of Case 3 in the right eye (a) and the left eye (b) FA revealed leakage from the optic disc and retinal arteries and veins in both eyes FA: fluorescein angiography

FAF revealed granular hyperfluorescence caused by pigment epithelial damage around the macula in both eyes. FA revealed leakage from the optic disc and retinal arteries and veins in both eyes, indicative of bilateral retinal vasculitis(Figure 5).

**Figure 6:**
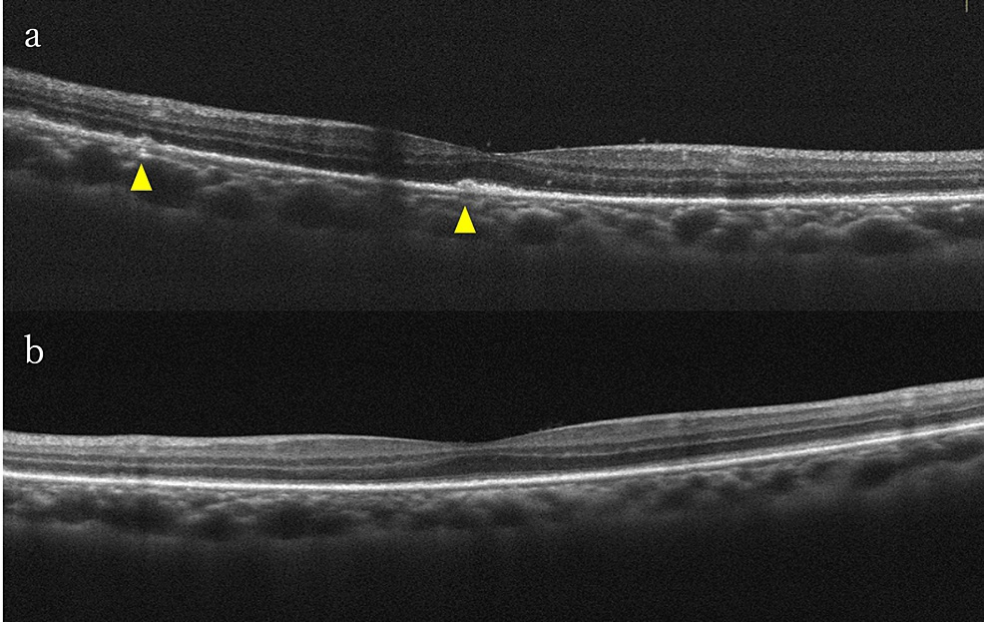
OCT findings in the right eye of Case 3 before and after intravenous antibiotic treatment (a) Pretreatment OCT showed depicted nodular retinal pigment epithelium irregularity (arrowheads). (b) Outer retinal findings had disappeared two months after treatment OCT: optical coherence tomography

OCT showed indistinct EZ and RPE in the right eye, along with punctate hyper-reflective lesions in the inner layers (Figure 6).

Relative afferent pupillary defect was obvious in the right eye and central flicker values were 18 Hz in the right eye and 36 Hz in the left eye, indicating optic neuropathy in the right eye. Laboratory tests were positive for RPR and TP antibodies. HIV, HBV, and HTLV-1 test results were negative. There were scars of 3-4 mm in diameter on the penis. Based on these findings, syphilitic uveitis was diagnosed. The patient was prescribed a 14-day course of intravenous PCG at 18 MU/day. The retinal vasculitis and hyperfluorescent area on FAF persisted, while the central flicker values normalized to 46.3 Hz in the right eye and 46.7 Hz in the left eye after treatment completion. There was no detection of a relative afferent pupillary defect. At 55 days after treatment completion, visual acuity improved to 20/40 in the right eye and 20/20 in the left eye. While FAF still revealed entire hyper-autofluorescence (Figure 4), OCT showed well-defined EZ and RPE (Figure 6). The central scotoma in the right eye also improved. The RPR quantitative test improved from 19,000-fold before treatment to 2,300-fold after treatment.

Case 4

**Figure 7:**
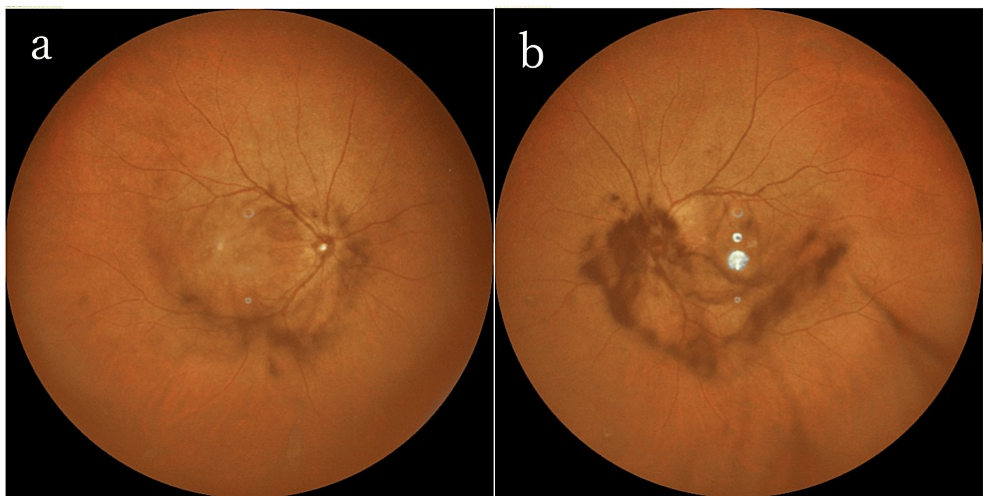
Color fundus photography of the right and left eyes at presentation in Case 4 Fundus examination showed grade 1+ vitreous haze in both eyes and vitreous hemorrhage in the left eye

A 47-year-old male visited an ophthalmologist complaining of decreased vision and floaters in his right eye for the past two months. He had a history of sexual encounters with sex workers and had a genital ulcer one year before, which had resolved without treatment after three months. He was diagnosed with bilateral uveitis and started on betamethasone eye drops for the right eye, resulting in improvement of his ocular symptoms. However, he returned to the clinic three months later due to deteriorating visual function, attributed to cystoid macular edema (CME) in both eyes. He was subsequently referred to Hiroshima University Hospital. At the initial visit, visual acuity was 20/50 in the right eye and 20/100 in the left eye. Intraocular pressure of the right and left eyes was 15 and 14 mmHg, respectively. The patient presented with grade 1+ anterior chamber cells and grade 1+ vitreous haze in both eyes. Vitreous hemorrhage was observed in the left eye (Figure 7).

**Figure 8:**
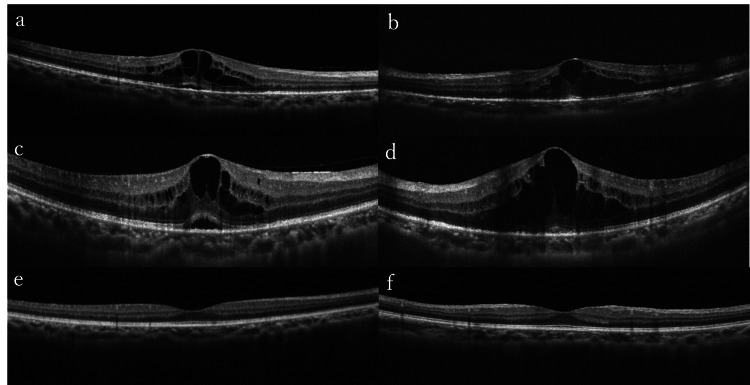
OCT of the right eye (a, c, e) and left eye (b, d, f) in Case 4 (a, b) OCT at the initial visit showed CME in both eyes and the subretinal fluid in the right eye. There were hyper-reflective dots in the inner retina and retinal pigment epithelium became irregular and hyper-reflective in both eyes. (c, d) CME worsening led to the initiation of betamethasone eye drops and oral prednisolone. Choroidal vessels were dilated. (e, f) Five months after treatment completion, OCT showed no abnormality in both eyes CME: cystoid macula edema; OCT: optical coherence tomography

No optic disc swelling, hyperemia, or yellow-white lesions were observed. OCT showed CME in both eyes, RPE irregularity, and punctate hyper-reflective dots in the inner layer. Serous retinal detachment was also present in the right eye (Figure 8).

**Figure 9:**
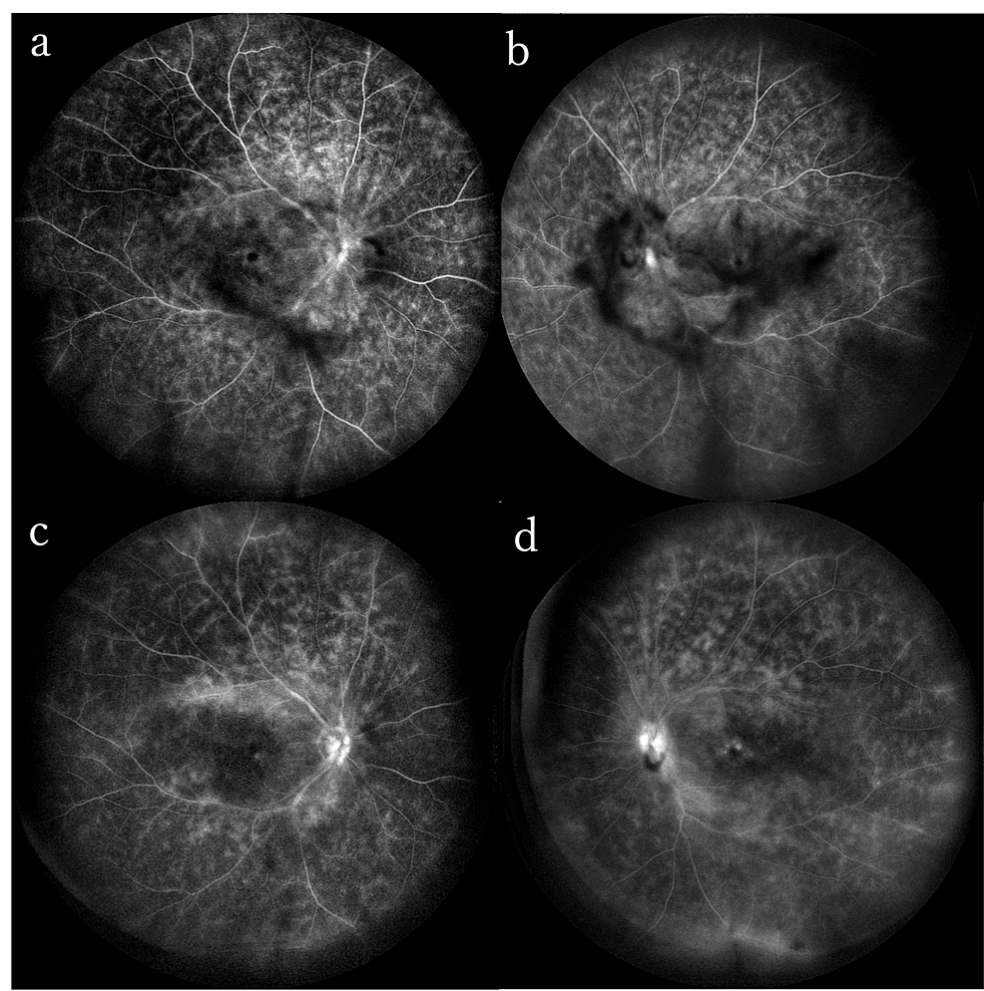
FA before and after treatment in Case 4 (a, b) FA of the right and left eyes at presentation showed fern-like leakage from capillaries of all quadrants and optic disc hyperfluorescence. A vitreous haze around the disc blocked the leakage. (c, d) FA of the right and left eyes four months after antibiotic treatment. Although vitreous haze was improved, vasculitis was still evident FA: fluorescein angiography

FA revealed fern-like fluorescence leakage from whole retinal capillaries in both eyes and leakage from the optic disc (Figure 9). FAF showed hypofluorescence around the macula. The central flicker test results were as follows: 37 Hz in the right eye and 41 Hz in the left eye. RPR and anti-TP antibody tests were positive, while HIV, HBV, HTLV-1, and other infection tests were negative. Based on ocular findings and laboratory tests, syphilitic uveitis in both eyes was diagnosed, and intravenous PCG (24 MU/day) was administrated. Five days after starting antibiotic therapy, anterior chamber cells decreased to grade 0.5+, and vitreous haze improved in both eyes. However, 11 days after the initiation of PCG, vitreous haze recurred and CME worsened bilaterally on OCT (Figure 8), leading to the initiation of betamethasone sodium phosphate eye drops for both eyes and oral prednisolone 25 mg/day, tapered over one month. Two months after treatment, visual acuity improved to 20/20 in the right eye and 20/25 in the left eye, and the RPR quantitative test showed a decrease from 162-fold to 72.6-fold. Five months after treatment, vitreous haze, CME, and irregularity of the RPE were no longer evident (Figure 7). However, persistent fern-like fluorescence leakage was observed in both eyes, with the patient reporting blurred vision (Figure 9).

As the RPR quantitative test showed no signs of syphilis recurrence, systemic corticosteroid therapy at 30 mg/day was resumed and subsequently tapered off upon resolution of the fern-like fluorescence leakage.

## Discussion

Syphilis affects every structure of the eye, earning it the nickname “the great imitator” as it mimics various diseases [4]. Diagnosis of syphilis is often challenging, and delay in appropriate antibiotic treatment can lead to poor visual prognosis [5]. It is not uncommon for patients to be misdiagnosed with noninfectious uveitis and initiated on steroid therapy, leading to temporary symptomatic improvement, which may worsen during steroid titration or termination [4,8-9]. According to Parthopratim, the frequency of syphilitic uveitis by location is as follows: anterior uveitis: 10%, posterior uveitis: 13%, optic neuritis: 22%, panuveitis: 41%, and vitreous inflammation: 65% [5]. One report suggests that 90% of cases exhibit lesions in the posterior segment of the eye [10]. In our case series, one patient had anterior uveitis and three had panuveitis, with two cases also presenting with optic neuropathy. Acute syphilitic posterior placoid chorioretinitis, as seen in Case 2, is a specific but rare manifestation of ocular syphilis [11].

Ocular syphilis can occur at any stage of syphilis and be associated with neurosyphilis [12]. According to medical history and genital condition, all of our cases were secondary syphilis. None of our patients had undergone cerebrospinal fluid testing or MRI scans. As per Centers for Disease Control and Prevention guidelines, for patients with isolated ocular symptoms (no cranial nerve dysfunction or other neurologic abnormalities), reactive syphilis serology, and confirmed ocular abnormalities on examination, cerebrospinal fluid examination is unnecessary before treatment [12]. Given that cerebrospinal fluid laboratory abnormalities are common among persons with early syphilis [12], it would have been preferable to perform a cerebrospinal fluid examination.

While the pathogenesis of syphilitic uveitis is unclear, some hypotheses suggest direct invasion and secondary obstruction of the choriocapillaris by spirochetes, and deposition of soluble immune complexes in the tissues [11]. In acute syphilitic posterior placoid chorioretinitis, irregular hyper-reflectivity of the RPE with nodular elevations corresponds to hyperfluorescent dots within the yellow-white discoid lesions on FAF, indicating lipofuscin accumulation and incomplete phagocytosis of retinal photoreceptor cell outer segment [1,13].

OCT is a valuable tool for diagnosing and monitoring ocular syphilis involving the retina and choroid. In a study by Vaze et al., all 54 eyes with syphilitic uveitis involving the posterior segment had at least one retinal finding on OCT [14]. The findings in the outer retina always involve RPE changes, such as thickening, irregularities, detachment, and hyper-reflective nodular elevations [14]. RPE and outer retinal lesions caused by syphilis can be reconstituted after antibiotic treatment [15]. RPE findings are reversible and vision improves in most cases, but prolonged outer retinal layer lesions, especially EZ disruption, have been associated with poor visual prognosis [1]. In our patients, six eyes (75%) showed outer retinal involvement; all resolved following antibiotic treatment. Furthermore, five eyes demonstrated improvement in best-corrective visual acuity to more than 20/25.

While OCT provides a detailed view of outer retinal layers and RPE damage and recovery with treatment, it can only assess a limited range of the retina. In contrast, FAF enables the evaluation of RPE damage and recovery over a broader area. It has been reported that hyperfluorescence of FAF becomes normal along with the resolution of placoid lesions and partial or complete reconstitution of outer retinal layers [15]. Additionally, FAF facilitates the detection of subtle fundus changes in initial acute syphilitic posterior placoid chorioretinitis, thereby contributing to early diagnosis [16].

FA plays an important role in evaluating elusive vasculitis and optic disc inflammation [17]. Klein reported that 78% of cases of ocular syphilis had optic nerve involvement [18]. Even in cases that seem to have only anterior inflammation, as in Case 1 in our series, FA can detect optic disc leakage and mild vasculitis. Balaskas et al. demonstrated a high rate of disappearance of vascular staining in FA, suggesting that vasculitis in ocular syphilis may not lead to visual consequences [19]. However, vasculitis may persist despite antibiotic treatment, as in Case 4 in our series. FA can be performed after the completion of antibiotic treatment if the patient complains of ocular symptoms, such as blurred vision or floaters, to detect remaining retinal vascular inflammation and to determine if additional treatment is necessary. In Case 4, after serological tests confirmed that there was no reinfection, the patient was treated with steroid monotherapy again and the vasculitis improved.

## Conclusions

We discussed our experience with four cases of syphilitic uveitis with varying presentations; we were able to establish accurate diagnoses and assess treatment efficacy by using multimodal imaging tools such as OCT, FA, and FAF, in addition to syphilitic serological testing. Ocular syphilis is associated with diverse clinical manifestations, and delayed diagnosis can result in irreversible damage. Whenever uveitis is encountered, especially with outer retinal findings, syphilis should be considered in the differential diagnosis, and appropriate treatment should be guided by a combination of various ophthalmological examinations.
